# Broad spectrum microarray for fingerprint-based bacterial species identification

**DOI:** 10.1186/1472-6750-10-13

**Published:** 2010-02-17

**Authors:** Frédérique Pasquer, Cosima Pelludat, Brion Duffy, Jürg E Frey

**Affiliations:** 1 Agroscope Changins-Wädenswil Research Station ACW, Laboratory for Molecular Diagnostics and Epidemiology, CH-8820 Wädenswil, Switzerland; 2 Agroscope Changins-Wädenswil Research Station ACW, Phytopathology Group CH-8820 Wädenswil, Switzerland

## Abstract

**Background:**

Microarrays are powerful tools for DNA-based molecular diagnostics and identification of pathogens. Most target a limited range of organisms and are based on only one or a very few genes for specific identification. Such microarrays are limited to organisms for which specific probes are available, and often have difficulty discriminating closely related taxa. We have developed an alternative broad-spectrum microarray that employs hybridisation fingerprints generated by high-density anonymous markers distributed over the entire genome for identification based on comparison to a reference database.

**Results:**

A high-density microarray carrying 95,000 unique 13-mer probes was designed. Optimized methods were developed to deliver reproducible hybridisation patterns that enabled confident discrimination of bacteria at the species, subspecies, and strain levels. High correlation coefficients were achieved between replicates. A sub-selection of 12,071 probes, determined by ANOVA and class prediction analysis, enabled the discrimination of all samples in our panel. Mismatch probe hybridisation was observed but was found to have no effect on the discriminatory capacity of our system.

**Conclusions:**

These results indicate the potential of our genome chip for reliable identification of a wide range of bacterial taxa at the subspecies level without laborious prior sequencing and probe design. With its high resolution capacity, our proof-of-principle chip demonstrates great potential as a tool for molecular diagnostics of broad taxonomic groups.

## Background

Microarray platforms for practical diagnostics have been developed for identification of human/animal- and plant-pathogenic species. These have been primarily designed as taxa-specific chips, and have required extensive knowledge of sufficiently discriminatory genes such as the cytochrome oxidase 1 (CO1) or the cytochrome b genes for insects and mammals [1], 16 S rDNA, *rpoB*, *groEL *or *gyrB *for bacteria [2-4], or coat-protein genes for viruses [5]. Probe selection with adequate specificity to identify organisms at the genus, species, and less frequently subspecies (e.g., serotypes, pathovars) entails labour- and time-intensive sequencing. Substantial effort and resources must be invested to compensate for inherent intra-group genetic variation of diagnostic DNA fragments, demanding sequencing of a representative number of individual organisms or isolates in order to detect all potential variations [6]. Diagnostics in clinical, animal or plant health demand exclusion of a broad-spectrum of unknown but related taxa that are inconsequential for therapeutic or regulatory purposes, while rapidly and sensitively detecting select-agent and/or pathogenic organisms. Achieving identification at a high level of taxonomic resolution such as strains and/or pathovars typically requires incorporation of multiple target regions, each of which requires individual design and validation steps [4].

Inadequacy of available sequence information for many taxa of interest poses a fundamental problem in probe selection during diagnostic microarray design. A panel of candidate probes must be evaluated by trial-and-error since most do not hybridise as anticipated. Even though many probes hybridise to correct target sequences, often hybridisation is weak or produces stronger signals for mismatch targets [7,8]. In gene expression studies using chips carrying oligonucleotide probes that target thousands of genes, this aspecificity phenomenon is usually masked by the massive data output, and thus it has only recently received attention [9-11]. This problem was identified earlier with diagnostic chip analysis since these carry far fewer probes than transcriptomic microarrays. False hybridisations on diagnostic chips result in a lack of specificity and incorrect identifications. Hybridisation performance of probes cannot be predicted accurately, therefore, multiple (i.e., three to five) as opposed to single candidate probes must be designed for each target, each requiring evaluation of specificity 'on-chip', in order to validate performance reliability.

With few exceptions, diagnostic microarrays have thus far been developed for identification of organisms within narrow taxonomic groups (i.e., within a family or genus) or are limited to a few organisms that can be expected to occur on a single host. Our objective in this study was to develop a broad spectrum chip that would facilitate identification of bacteria regardless of taxa and without requiring *a priori *knowledge of sequence targets, based solely on complete genome hybridisation patterns. The high-density chip we designed incorporates short oligonucleotide probes of random sequence, and requires neither *a priori *sequence information nor species-specific probe design or and validation. A database comprised of hybridisation type-patterns for reference target organisms is required, as has been established for other 'gold-standard' diagnostic technologies, but no sequencing is needed throughout the entire process. Species identification will then be assessed based upon match of their hybridisation pattern with this pre-established taxon-specific database. Thus, this strategy does not allow unambiguous identification of taxa not present in the database. Our chip carries 95,000 unique 13-mer probes assembled on-chip by NimbleGen^® ^System Inc. This large number of small oligomers theoretically enables hybridisation of circa 10,000 probes per bacterial genome having a few Mb genome size. Performance and reliability of this chip applied for discrimination of bacteria at the species and strain level is presented.

## Results

We set out to develop a genome chip for discrimination of bacteria without prior knowledge of gene targets or specific probe design. Our aim was to obtain reproducible hybridisation patterns specific at the deepest possible taxonomic level (i.e., genus, species, and subspecies).

**Figure 1:**
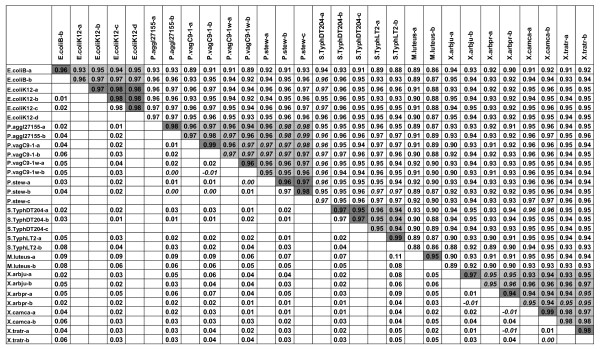
**Correlation coefficients between hybridisation patterns obtained with all 95,000 probes**. Spearman's rank correlation coefficients (top right) and difference between mean of replicate pair-wise correlations of one isolate to mean of non replicate pair-wise correlations of other isolate, both reciprocal values are indicated below each other (down left). Reproducibility is demonstrated through the observed correlation values which are higher between the replicates than with samples from other species for most of the cases. Values from the same genera are highlighted in light grey and replicates from the same strains with a darker grey. Correlation can be higher between samples from different strain or species (indicated in italics) than between replicates, revealing the need of sorting out aspecific probes. P. aggl stands for *P. agglomerans*, P.vag for *P. vagans*, P. stew for *P. stewartii *subsp. *indologenes*, S. Typh for *Salmonella *Typhimurium, X.arbju for *X*. *arboricola *pv. *juglandis*, X.arbpr for *X. arboricola *pv. *pruni*, X.camca for *X. campestris *pv. *campestris *and X.tratr for *X. translucens *pv. *translucens*. Replicates are indicated from a to d.

Hybridisations were performed at low temperature (4°C) which enhanced reproducible probe capture of specific targets, similar to a previous report with a short-oligonucleotide diagnostic microarray for differentiation of *Xanthomonas citri *pathovars [12]. Although the low hybridisation temperature might increase the number of false positive hybridisation (i.e., to probes with an imperfect match), our results were highly reproducible as demonstrated by high correlation coefficients between replicates (Figure 1). These values range from 0.95 up to 0.99 between strain replicates (average correlation values of 0.97 +/- 0.002 standard error) and were generally lower between different strains/species (down to 0.86 between *M. luteus *and *Salmonella *Typhimurium LT2 or *Escherichia coli *strain B samples). This demonstrated that species and even strain dependent hybridisation patterns were obtainable. In fact, there were more hybridised probes observed than expected based on the assumption that hybridisation would occur only on perfect-match probes with small genome organisms such as bacteria. This indicates that hybridisation also occurred with probes containing mismatches.

### Hybridisation behaviour of perfect-match probes

To assess the hybridisation behaviour of perfect match probes, we studied the *E. coli *K12, and *Salmonella *LT2 strains whose genomes have been sequenced [13,14]. *E. coli *K12 DNA (4.5 Mb genome plus a 5-kb plasmid) showed a perfect match to 10,815 probes in BLAST analysis, and *Salmonella *LT2 (4.8 Mb genome) to 9,849 probes. This corresponds to the expected coverage toward bacterial genomes of oligomers with random probe sequence. Closer study on the hybridisation behaviour of these genome-specific probe sets showed a broad range from no or low-level hybridisation to saturated hybridisation levels (65,535) for either the *E. coli *K12 samples or the *Salmonella *LT2 samples. Since most of these probes had only one perfect match on their respective genome, there was no bias induced by the frequency of the sequence in the genome on the hybridisation level. Unexpectedly, a large number of probes not belonging to the perfect match sets were showing high hybridisation signals. Thus, while some targets do not hybridise at all to perfect match probes, others hybridise to mismatch probes. In fact we found that irrespective of the applied background noise level, only 12-17% of all hybridised probes were perfect match probes and the remaining were mismatch probes. For example, using the 10,000 lowest hybridisation intensity probes that do not occur in the *E. coli *K12 genome as background level, we found 8,567 *E. coli *K12 perfect match probes with a positive hybridisation signal. The total number of positive hybridisation signals was 70,667 probes, indicating a large fraction of mismatch hybridisation. However, our results provide strong evidence that mismatch hybridisation was as reproducible as full match hybridisation. For our strategy, mismatch hybridisation therefore delivers the same information content as perfect match hybridisation, and contributes to the discrimination power of the assay.

Similar hybridisation results were obtained using the *E. coli *K12 perfect-match probe set with *E. coli *B, *E. coli *K12, *P. agglomerans*, *P. vagans *and *P. stewartii *strains, and *X. arboricola *pathovars, as reflected by high correlation values (Additional file 1) and low class prediction percentage of 73.3% (Table 1). No discrimination was possible apart from the *Salmonella *DT204 and LT2, *X. campestris *and *translucens *and *M. luteus *replicates. Stronger hybridisation signals were even obtained with this probe set for strains different from *E. coli *K12, including the Gram-positive strain *M. luteus*. Similar hybridisation results and low class prediction (CP) percentage (76.6%) were obtained with the *Salmonella *LT2 probe set (Table 1).

**Table 1:** Class prediction based evaluation of discrimination correctness expressed as percentage of the probe sets that were determined by one-way ANOVA with Benjamini and Hochberg false discovery rate (FDR) and of the probe sets corresponding to perfect match with *E. coli *K12 and *Salmonella *LT2 genomes.

Probe sets (FDR *P*-value)	Number of probes	Correct predictions	Percentage of correct predictions
complete	95,000	23/30	76.6%
0.05	69,811	25/30	83.3%
0.01	46,858	29/30	96.6%
0.005	38,220	29/30	96.6%
0.001	21,265	29/30	96.6%
0.0005	15,439	29/30	96.6%
0.0003	12,071	30/30	100%
0.0001	6,379	30/30	100%
0.00005	4,062	30/30	100%
0.00001	1,160	30/30	100%
0.000005	507	29/30	96.6%
K12-match	10,895	22/30	73.3%
LT2-match	9,849	23/30	76.6%

### Hybridisation behaviour of the complete probe set

With the complete probe set, correlation values between samples from the same species (e.g., *X. arboricola *pathovars) or from the same genera (*P. stewartii *subsp. *indologenes *and *P. agglomerans *strains) were sometimes higher than between strain replicates (Figure 1). This was reflected by the low CP percentage of 76.6% (Table 1). Due to the large number of probes, it was expected that many probes would hybridise across related species which likely explained the high similarity of patterns between close relatives. Therefore, species-specific hybridisation patterns should be defined without these aspecific probes.

### Evaluation of different discriminatory probe sets

**Figure 2:**
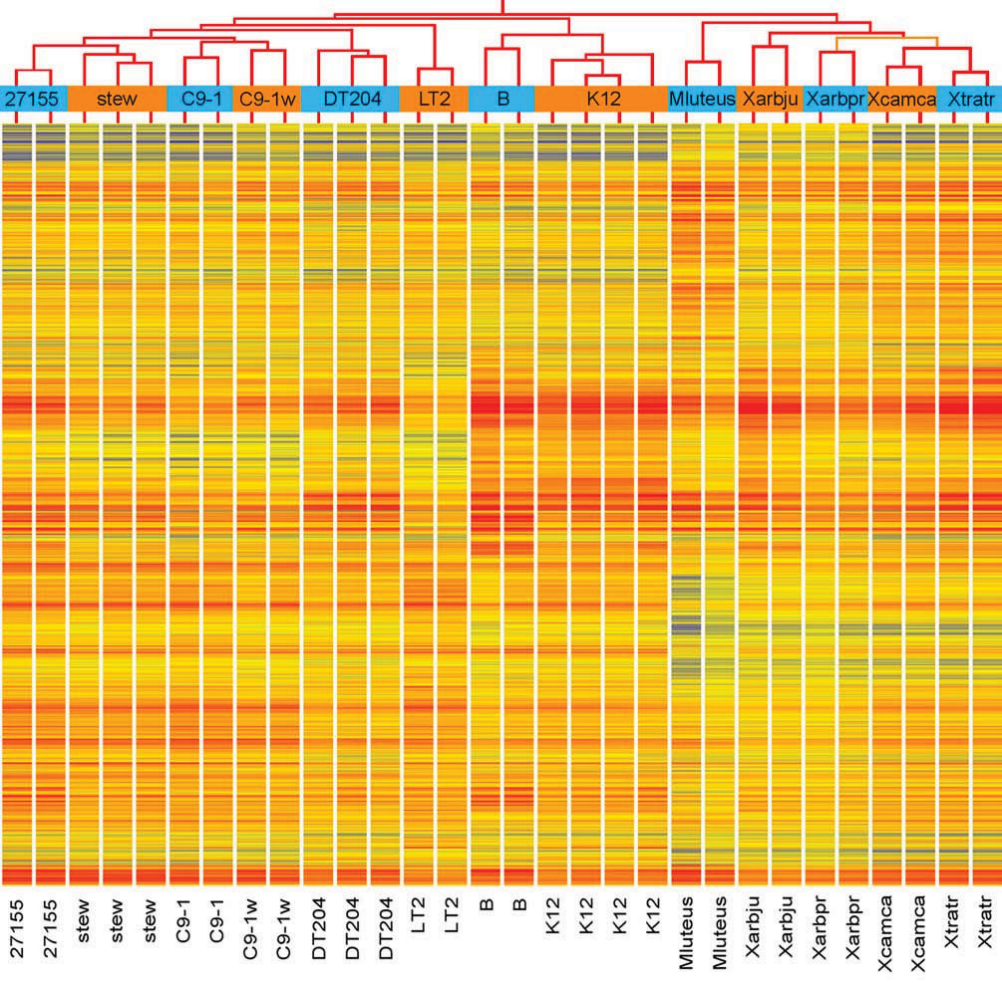
**Hybridisation patterns of 13 bacterial strains and variant for 12,071 probes selected by ANOVA with a *P*-value of 0.0003**. Clustering was performed with GeneSpring v7.3.1 (Agilent Technologies) on the replicates with Spearman's rank correlation (confidence level calculated with bootstrapping). stew:*P. stewartii *subsp. *indologenes *DC283, *P. agglomerans *strain ATCC27155^T^, C9-1: *P. vagans *strain C9-1, C9-1w: *P. vagans *C9-1w plasmid-cured derivative, DT204: *Salmonella *Typhimurium strain DT204, LT2: *Salmonella *Typhimurium strain LT2, B: *E. coli *strain B, K12: *E. coli *strain K12, Mluteus: *M. luteus*. Xarbju: *X. arboricola *pv. *juglandis*, Xarbpr: *X. arboricola *pv. *pruni*, Xcamca: *X. campestris *pv. *campestris*, Xtratr: *X. translucens *pv. *translucens*. Colour scale: blue: low normalised hybridisation value (N.H.V.), yellow: mid N.H.V., red: high N.H.V; red branches of the cluster represent 100% confidence, and orange: 74.4%.

**Figure 3:**
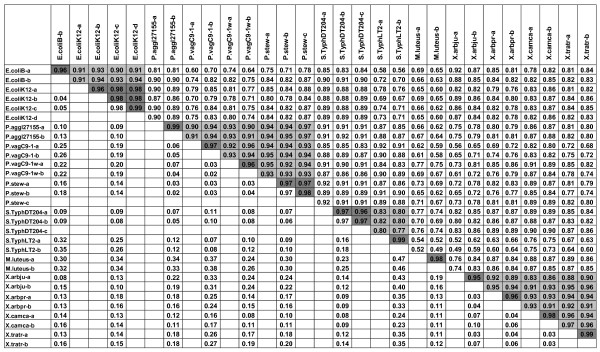
**Correlation coefficients of hybridisation patterns obtained with 12,071 probes selected by ANOVA with a *P*-value of 0.0003**. Spearman's rank correlation coefficients (top right) and difference between mean of replicate pair-wise correlations of one strain to mean of non replicate pair-wise correlations of another strain, both reciprocal values are indicated below each other (down left). Values from the same genera are highlighted in light grey and replicates from the same strains with a darker grey. Correlation coefficients between pair-wise replicates are higher than the ones between strains, species or genera. P. aggl represents *P. agglomerans*, P.vag for *P. vagans*, P. stew for *P. stewartii *subsp. *indologenes*, and S. Typh for *Salmonella *Typhimurium, X.arbju for *X*. *arboricola *pv. *juglandis*, X.arbpr for *X. arboricola *pv. *pruni*, X.camca for *X. campestris *pv. *campestris *and X.tratr for *X. translucens *pv. *translucens*. Replicates are indicated from a to d.

Taxon-specific hybridisation patterns (i.e., type-patterns), were established by entering prior information on group affiliation of the replicate hybridisation experiments into the data analysis software. Based on this prior information, we identified discriminatory probes showing similar hybridisation signals between slide replicates but differences between the thirteen species/strains/variants by means of ANOVA performed in GeneSpring GX v7.3.1. Decreasing *P*-values between 0.05 and 0.000005 were tested for the ANOVA using the Benjamini and Hochberg false discovery rate as a multiple testing correction procedure to obtain different probe sets. The use of this correction parameter increases the confidence that the obtained probe sets were selected due to high reproducibility of hybridisation results. Best discriminatory probe lists were then determined by cross-validation (K-nearest neighbour method with Fischer's exact test) with the class prediction analysis tool of GeneSpring GX v7.3.1. The percentage of correct prediction for the different probe selections ranges from 83.3-100% (Table 1). The probe sets obtained were finally used for grouping by cluster analysis based on Spearman's rank correlation with average linkage and confidence levels determined by boot-strapping. Using the first probe set (*P*-value of 0.05 corresponding to 69,811 selected probes providing 96.6% of correct class prediction, Table 1), the cluster obtained roughly corresponded to the expected hierarchical phylogeny. All *Enterobacteriacea *formed a large cluster from which the replicates of *Microccocacea M. luteus *and *Xanthomonas *pathovars were excluded. The two *E. coli *strains clustered together, as did the *Pantoea *species. However, one *P. stewartii subsp. indologenes *grouped together with the *P. vagans *C9-1 samples within the *Pantoea *sub-cluster instead of its respective strain sub-cluster and *X. arboricola *pv. *pruni *samples were not discriminated from the *X. arboricola *pv. *juglandis *ones (data not shown). The *Salmonella *strains DT204 and LT2 did not cluster together (although replicates within a strain did) but they clustered within the *Escherichia *group or to the *Pantoea *group, respectively. Similar results were obtained with *P*-values of 0.01 and 0.005 (96.6% CP, Table 1), but with the two *Salmonella *strains linked as independent sub-clusters to the *Pantoea *group. Correct clustering of each strain was obtained with a *P*-value of 0.001 (21,265 probes) but prediction reached only 96.6%; only the *P. agglomerans *strains and variants were not sub-clustering in the expected phylogenetic manner. Decreasing the *P*-value to 0.0003 (12,071 probes) resulted in 100% correct CP (Table 1), correct group allocation as shown in Figure 2 and high correlation coefficients between replicates (Figure 3). Similar CP results were obtained with *P*-values of 0.0001, 0.00005 and 0.00001 which all had prediction results of 100% (Table 1); only the two latter did not show the same clustering performance as the others as *X. arboricola *pv. *juglandis *could not be distinguished from *X. translucens *pv. *translucens *(data not shown). The sets obtained with *P*-values of 0.0003 and 0.0001 correspond to the optimal range for species, strain and variant discrimination for our strain panel as both correct class predictions and cluster representations were obtained. Lower performance for discriminating the *Xanthomonas *species were observed when decreasing the significance level to *P*-value of 0.000005 corresponding to 507 probes (Table 1). Hybridisation patterns of the *Pantoea *strains and *Xanthomonas *pathovars were highly similar to each other indicating the limitations of the chip at its current state of development and under the given conditions.

Finally, it is important to note that when ANOVA was performed based on random group allocation of taxa, no probe list could be determined that would discriminate these groups. This confirms that, despite the huge number of probes, the observed taxon-specific patterns were obtained because they reflected the underlying genomic sequences rather than just statistical noise.

## Discussion

We have developed a high-density genome chip and experimental conditions for identification of bacterial pathogens at the species and subspecies level, based on genomic hybridisation patterns. The main advantages of our strategy using a genome chip are that i) no *a priori *knowledge of the genome is required, ii) the genetic markers cover the entire genome, and iii) due to the large number of probes, the hybridisation patterns and hence the derived taxon identifications are very robust. This strategy could potentially be applied to a broad range of bacterial strains of health or economic interest, and extended to many living organisms. Our approach is a modification of a strategy originally developed in our laboratory since 1999 using many anonymous markers to fingerprint genomes [15]. The basic concept of generating many anonymous markers from a genome to produce a specific pattern was conceived in the early 1990 s and realized in the form of RAPD-PCR for species identification [16] and marker search [17,18]. Agarose gels were used to detect RAPD-PCR amplicons but this technique suffers from lack of reproducibility. With the advent of microarrays it became obvious that attempts should be made to exploit these new tools for fingerprint-based taxon characterisation. Eventually, a strategy similar to the one developed at our laboratory with a microarray containing 14,283 anonymous probes was successfully applied to identify bacterial and other genomes [19]. Together with the data shown here this supports our conclusion that fingerprint-based species identification is a powerful tool.

Observed hybridisation patterns were robust and highly reproducible, and they enabled differentiation at the deepest taxonomic level used in our experiments. For example, we achieved discrimination of strains within the same serovar (*Salmonella*), of different pathovars within the same species (*X. arboricola *pv. *juglandis *and pv. *pruni*) and at the subspecies level with a strain-variant cured of a single plasmid (*P. agglomerans*). Grouping of the different species was a close match to phylogenetic expectations. The problematic/non-phylogenetic positioning of *Salmonella enterica *Typhimurium strains using our chip may be explained by the genetic diversity observed within this serovar [20], which has recently been considered for differentiating *Salmonella *serovars into genovars [21]. However, phylogenetically "incorrect" clustering of strains did not interfere with taxon identification enabled by the determined probe sets with their high class prediction rates (Table 1).

Hybridisation patterns we obtained were not sequence specific *sensu stricto *(e.g., *E. coli *K12 DNA did not match exclusively to K12-specific probes). However, the hybridisation patterns were highly reproducible among replicates within the same strain. The off-target hybridisation phenomenon has been recently taken into account with oligonucleotide microarrays used for gene expression studies and can lead to incorrect interpretations of biological issues [10]. It is critical for diagnostic chips based on sequence-specific probes to eliminate such non-specific hybridisation. However, in our study, we took advantage of mismatch hybridisations by considering these probes as informative as perfect-match probes provided the results were reproducible between replicate samples.

## Conclusions

This work provides a foundation for building a fingerprint database containing important bacterial species that can be hybridised on this broad spectrum chip. Probes that do not give reliable results may eventually be discarded to streamline the chip design. However, sufficient probes have to be retained to ensure sufficient fingerprint polymorphism for other bacterial species to be identified, such as more Gram-positives which were only represented by *M. luteus *in our panel. This should eventually enable selection of a reduced probe set capable of comprehensive species and strain identification. In terms of strain fingerprinting and microbial source tracking, this genome chip offers a potential alternative to current technology based on RFLP, microsatellite or MLST approaches [22]. Starting with low amounts of genomic DNA followed by whole genome amplification could overcome problems of cultivation and facilitate the identification process, mostly when starting from unknown material.

## Methods

### Species, strains and pathovars used

Four Gram-negative *Enterobacteriaceae *species and one Gram-positive *Micrococcaceae *species were used to determine the reliability of our genome chip approach for taxon identification at the supra-species level. We selected the two *Escherichia coli *strains (K12 and B) because the K12 strain has been fully sequenced and is known to differ from the B strain by presence or absence of specific genomic regions. Two *S. enterica *Typhimurium strains (LT2 with available genome sequence, and DT204) were used. *Salmonella *strains are known for frequent lateral gene transfer [14,23] and complex genome variations [24,25], and are ideal targets for examining the serotype concept with microarray hybridisation profiling studies. Therefore, the concept of genovars is increasingly used to identify genotypes within serovar groups [21]. The *Pantoea *genera contain species of clinical, plant pathogenic and beneficial plant-protection nature. The type strain *P. agglomerans *ATCC 27155 was isolated from a knee laceration and *P. vagans *C9-1 is commercialized for biological control of plant diseases, such as fire blight caused by *Erwinia amylovora*. A variant of C9-1 lacking the 530 kb plasmid pPag3 (C9-1w) which carries pigmentation genes was isolated in our laboratory, and selected for determination at the sub-strain level on our chip. The related *Pantoea stewartii *subsp. *indologenes *strain DC283 is a plant pathogen of maize [26]. *Micrococcus luteus *was included as a representative of a Gram-positive bacterium with a high G-C content (65-75%), and because it is phylogenetically distant from the Enterobacteriacea used. Plant pathogen bacteria from the Xanthomonadaceae family (*Xanthomonas arboricola *pv *juglandis *CFBP 7179, *X. arboricola *pv *pruni *CFBP 5530, *X. translucens *pv *translucens *CFBP 2054 and *X. campestris *pv *campestris *CFBP 5241) were included to test the determination of our chip at the pathovar level [27,28]. Strains used in the study originated from the French Collection of Phytopathogenic Bacteria (collection française de bactéries phytopathogènes, CFBP strains), the American Type Culture Collection (ATCC strain), University of Wisconsin-Madison (*E. coli *K12) the Robert Koch Institut, Wernigerode, Germany, Institute of Public Health, Research Laboratory for Infectious Disease (*Salmonella *Typhimurium LT2 and DT204), Oregon State University, V.O. Stockwell, (*P. stewartii *subsp. *indologenes *DC283, *P. vagans *C9-1 (*P. vagans *C9-1w in house)), and were taxonomically verified in our laboratory. Strain C9-1 previously *P. agglomerans *has been recently assigned to *P. vagans *[29-31].

### DNA extraction and labelling

Genomic bacterial DNA was extracted following the standard protocol described in Maniatis and Sambrook [32] (strains K12, ATCC27155^T^, C9-1, C9-1w, DC283, LT2, DT204, CFBP 7179, CFBP 5530, CFBP 2054 and CFBP 5241), or was commercially obtained (*E. coli *strain B and *M. luteus*, Sigma-Aldrich, Buchs, Switzerland). DNA was sonicated in a Bioruptor (Diagenode, Liege, Belgium) to obtain fragments of approximately 500 bp. Sonicated DNA (10 μg) together with 5 μl random hexamer 1 mM (Fermentas, Lab Force, Nunningen, Switzerland) were denatured 5 min at 95°C and cooled. Labelling reactions were performed by incubation overnight at 37°C in 50 μl with 40 U of Klenow fragment (Fermentas), 5 μl of 10 × buffer, 1 μl of Cyanine-3 labelled dUTP (Perkin Elmer, Schwerzenbach, Switzerland), 0.5 mM of dNTP (without dTTP) (Fermentas). Samples were purified using PCR purification kit (Qiagen, Basel, Switzerland). Quality and concentration was determined using a ND-1000 spectrophotometer (NanoDrop^®^, Witec AG, Littau, Switzerland).

### Nimblechip™ arrays

Several visual basic macros were written to design 13-bp long probes. Probes were generated by random selection among one of the four bases (i.e., A, C, T, G), adding the newly selected base to the growing oligonucleotide up to a length of 13 bases. Each new probe was added to a probe set after being checked for identity against all previously designed probes. All probes that were either identical or complementary to previously stored probes were discarded. Probes showing hairpin structures of more than 3-bp length were also discarded. Finally, the probes were selected for a minimal weighted difference approximately equivalent to one centrally located base pair by allocating difference scores based on the mismatch position. Positions 1 or 13 were given a score of 0.1, position 2 or 12 one of 0.2, 3 or 11 one of 0.3, 4 or 10 one of 0.5, 5 or 9 one of 0.7 and the remaining mismatches at positions 6, 7 and 8 obtained a score of 1.0. The mismatch scores were summed and all probes with a total score below 1.0 compared to the previously stored probes were discarded. With increasing numbers of probes this procedure progressively slows down the probe design process and therefore, the maximum number of probes per run was set to 37,000 and the macro was run three times independently to generate three sets of probes. Only 118 probes were found to be identical and/or complementary among these three probe sets indicating that probe generation was indeed random. These 118 redundant probes were discarded and the remaining 110,882 probes were merged into a single file. These probes were then checked for shifted homology, i.e., for probes that were identical if shifted by one to three base pairs. This procedure eliminated another 15,601 probes resulting in 95,281 unique probes of which the last 281 were discarded to form the final set of 95,000 probes.

Our custom chip contained four replicates of 95,000 probes synthesized on silanized glass slides [33,34]. The probes had two specific parts: a poly-dT 12-mer tail, linked to the chip surface by an amino-linker, and a 13-mer part of random sequence. Sets of 10,895 and 9,849 perfect-match probes were found to correspond to the *E. coli *K12 and *S. enterica *Typhimurium LT2 genome sequences, respectively, by blasting the entire probe set against their genome and plasmid sequences.

### Hybridisation

Prior to hybridisation, 3 μg of Cy3-labelled DNA in 100 μl of 2 × hybridisation buffer (NimbleGen^® ^System, Inc., Madison, WI, USA) and 40 μl Hybridisation Component A (NimbleGen^® ^System, Inc.), 0.3 μl of 3' labelled Cy3-CPK6 50 nM (Integrated DNA Technologies, Coralville, IA, USA) and ddH_2_O up to 200 μl were denatured for 5 min at 95°C then cooled. Hybridisation was performed over 16 hrs at 4°C with a pump rate of 1ml/min for preventing the risks of precipitation in an aHyb™ hybridisation station (Miltenyi Biotec, Bergisch-Gladbach, Germany). Washings were performed at 20°C in the station with NimbleGen^® ^Buffers I to III for 1 min each. Slides were incubated in the last washing buffer for 1 min before blow-drying with high-pressure air. Hybridisations were performed for four samples of *E. coli *K12, two *E. coli *B, two *S. enterica *Typhimurium LT2, three *S. enterica *Typhimurium DT204, three *P. stewartii *subsp. *indolognes *DC283, two *P. vagans *C9-1, two *P. vagans *C9-1w, two *P. agglomerans *ATCC27155^T^, two *M. luteus*, two *X. arboricola *pv. *pruni*, two *X. arboricola *pv. *juglandis*, two *X. campestris *pv. *campestris *and two *X. translucens *pv. *translucens*.

### Data analysis

Slides were scanned at a gain of 500-600 at 532 nm wavelength and 5-μm resolution with a Genepix 4100A scanner (Axon Instrument, Sunnyvale, California, United States of America). Quantification of hybridisation signal was performed with NimbleScan™ software (NimbleGen^® ^System, Inc.). Signals of each slide were smoothed using the NMPP program [35]. Normalisation per chip to 50th percentile and further analyses were performed in GeneSpring GX v7.3.1 (Agilent Technologies, Basel, Switzerland). One-way ANOVA was performed with different *P*-values to determine groups of probes with the same hybridisation pattern between replicates and giving the best discrimination of the species and subspecies. Class prediction analysis was used with the K-nearest neighbour method and Fisher's exact test to control the quality of ANOVA probe selections for identification of taxa. Reproducibility of the hybridisation results was tested at several technical levels: DNA extraction, sonication of DNA and labelling did not reveal any bias. The data discussed in this publication have been deposited in NCBI's Gene Expression Omnibus [36] and are accessible through GEO Series accession number GSE15391 http://www.ncbi.nlm.nih.gov/geo/query/acc.cgi?acc= GSE15391.

## Authors' contributions

FP conducted the microarray design, performed the experiments, analyzed the data and wrote the manuscript. CP participated in the microarray design and in conducting the experiments. BD and JEF conceived of and supervised the project and participated in writing the manuscript. All authors read and approved the final manuscript.

## Supplementary Material

Additional file 1 Pair-wise correlation coefficients for hybridisation patterns obtained with 10,895 probes corresponding to *E. coli *K12 perfect matches. Click here for file

## References

[B1] Pfunder M Holzgang O Frey JE Development of microarray-based diagnostics of voles and shrews for use in biodiversity monitoring studies, and evaluation of mitochondrial cytochrome oxidase I vs. cytochrome b as genetic markers Molecular Ecology 2004 13 5 1277 1286 10.1111/j.1365-294X.2004.02126.x 15078463

[B2] Loy A Schulz C Lucker S Schopfer-Wendels A Stoecker K Baranyi C Lehner A Wagner M 16 S rRNA gene-based oligonucleotide microarray for environmental monitoring of the betaproteobacterial order "Rhodocyclales" Appl Environ Microbiol 2005 71 3 1373 1386 10.1128/AEM.71.3.1373-1386.2005 15746340 PMC1065177

[B3] Kostic T Weilharter A Rubino S Delogu G Uzzau S Rudi K Sessitsch A Bodrossy L A microbial diagnostic microarray technique for the sensitive detection and identification of pathogenic bacteria in a background of nonpathogens Analytical Biochemistry 2007 360 2 244 254 10.1016/j.ab.2006.09.026 17123456

[B4] Sessitsch A Hackl E Wenzl P Kilian A Kostic T Stralis-Pavese N Sandjong BT Bodrossy L Diagnostic microbial microarrays in soil ecology New Phytol 2006 171 4 719 735 10.1111/j.1469-8137.2006.01824.x 16918544

[B5] Desantis TZ Stone CE Murray SR Moberg JP Andersen GL Rapid quantification and taxonomic classification of environmental DNA from both prokaryotic and eukaryotic origins using a microarray FEMS Microbiol Lett 2005 245 2 271 278 10.1016/j.femsle.2005.03.016 15837382

[B6] Meyer CP Paulay G DNA barcoding: error rates based on comprehensive sampling PLoS Biol 2005 3 12 e422 10.1371/journal.pbio.0030422 16336051 PMC1287506

[B7] Pozhitkov A Noble PA Domazet-Loso T Nolte AW Sonnenberg R Staehler P Beier M Tautz D Tests of rRNA hybridization to microarrays suggest that hybridization characteristics of oligonucleotide probes for species discrimination cannot be predicted Nucleic Acids Res 2006 34 9 e66 10.1093/nar/gkl133 16707658 PMC1463897

[B8] Binder H Preibisch S Kirsten T Base pair interactions and hybridization isotherms of matched and mismatched oligonucleotide probes on microarrays Langmuir 2005 21 20 9287 9302 10.1021/la051231s 16171364

[B9] Lee I Dombkowski AA Athey BD Guidelines for incorporating non-perfectly matched oligonucleotides into target-specific hybridization probes for a DNA microarray Nucleic Acids Res 2004 32 2 681 690 10.1093/nar/gkh196 14757833 PMC373323

[B10] Casneuf T Peer Y Van de Huber W In situ analysis of cross-hybridisation on microarrays and the inference of expression correlation BMC Bioinformatics 2007 8 461 10.1186/1471-2105-8-461 18039370 PMC2213692

[B11] Tembe W Zavaljevski N Bode E Chase C Geyer J Wasieloski L Benson G Reifman J Oligonucleotide fingerprint identification for microarray-based pathogen diagnostic assays Bioinformatics 2007 23 1 5 13 10.1093/bioinformatics/btl549 17068088

[B12] Kingsley MT Straub TM Call DR Daly DS Wunschel SC Chandler DP Fingerprinting closely related *Xanthomonas *pathovars with random nonamer oligonucleotide microarrays Appl Environ Microbiol 2002 68 12 6361 6370 10.1128/AEM.68.12.6361-6370.2002 12450861 PMC134374

[B13] Blattner FR Plunkett G III Bloch CA Perna NT Burland V Riley M Collado-Vides J Glasner JD Rode CK Mayhew GF The complete genome sequence of *Escherichia coli *K-12 Science 1997 277 5331 1453 1462 10.1126/science.277.5331.1453 9278503

[B14] McClelland M Sanderson KE Spieth J Clifton SW Latreille P Courtney L Porwollik S Ali J Dante M Du F Complete genome sequence of *Salmonella enterica *serovar Typhimurium LT2 Nature 2001 413 6858 852 856 10.1038/35101614 11677609

[B15] Frey JE Method for the characterizaion and/or identification of genomes Switzerland 2002 WO/2002/022870

[B16] Frey JE Frey B Molecular identification of six species of scales (*Quadraspidiotus *sp.) by RAPD-PCR: Assessing the field-specificity of pheromone traps Mol Ecol 1995 4 777 780 10.1111/j.1365-294X.1995.tb00279.x

[B17] Williams JG Kubelik AR Livak KJ Rafalski JA Tingey SV DNA polymorphisms amplified by arbitrary primers are useful as genetic markers Nucleic Acids Res 1990 18 22 6531 6535 10.1093/nar/18.22.6531 1979162 PMC332606

[B18] Welsh J McClelland M Fingerprinting genomes using PCR with arbitrary primers Nucleic Acids Res 1990 18 24 7213 7218 10.1093/nar/18.24.7213 2259619 PMC332855

[B19] Belosludtsev YY Bowerman D Weil R Marthandan N Balog R Luebke K Lawson J Johnson SA Lyons CR O'Brian K Organism identification using a genome sequence-independent universal microarray probe set BioTechniques 2004 37 654 660 15517977 10.2144/04374RR02

[B20] Scaria J Palaniappan RU Chiu D Phan JA Ponnala L McDonough P Grohn YT Porwollik S McClelland M Chiou CS Microarray for molecular typing of *Salmonella enterica *serovars Mol Cell Probes 2008 22 4 238 243 10.1016/j.mcp.2008.04.002 18554865 PMC2766089

[B21] Porwollik S Boyd EF Choy C Cheng P Florea L Proctor E McClelland M Characterization of *Salmonella enterica *subspecies I genovars by use of microarrays J Bacteriol 2004 186 17 5883 5898 10.1128/JB.186.17.5883-5898.2004 15317794 PMC516822

[B22] Santo Domingo JW Sadowsky MJ Microbial Source Tracking 2007 Washington D.C., USA: American Society for Microbiology Press

[B23] Brown EW Mammel MK LeClerc JE Cebula TA Limited boundaries for extensive horizontal gene transfer among *Salmonella *pathogens Proc Natl Acad Sci USA 2003 100 26 15676 15681 10.1073/pnas.2634406100 14671318 PMC307627

[B24] Porwollik S Wong RM McClelland M Evolutionary genomics of *Salmonella *: gene acquisitions revealed by microarray analysis Proc Natl Acad Sci USA 2002 99 13 8956 8961 10.1073/pnas.122153699 12072558 PMC124405

[B25] Pelludat C Prager R Tschape H Rabsch W Schuchhardt J Hardt WD Pilot study to evaluate microarray hybridization as a tool for *Salmonella enterica *serovar Typhimurium strain differentiation J Clin Microbiol 2005 43 8 4092 4106 10.1128/JCM.43.8.4092-4106.2005 16081956 PMC1233888

[B26] Coplin DL Frederick RD Majerczak DR Haas ES Molecular cloning of virulence genes from *Erwinia stewartii *J Bacteriol 1986 168 2 619 623 3782017 10.1128/jb.168.2.619-623.1986 PMC213525

[B27] Parkinson N Aritua V Heeney J Cowie C Bew J Stead D Phylogenetic analysis of *Xanthomonas *species by comparison of partial gyrase B gene sequences Int J Syst Evol Microbiol 2007 57 Pt 12 2881 2887 10.1099/ijs.0.65220-0 18048743

[B28] Vauterin L Rademaker J Swings J Synopsis on the taxonomy of the genus *Xanthomonas *Phytopathology 2000 90 7 677 682 10.1094/PHYTO.2000.90.7.677 18944485

[B29] Rezzonico F Smits TH Montesinos E Frey JE Duffy B Genotypic comparison of *Pantoea agglomerans *plant and clinical strains BMC Microbiol 2009 9 204 10.1186/1471-2180-9-204 19772624 PMC2764716

[B30] Brady C Cleenwerck I Venter S Vancanneyt M Swings J Coutinho T Phylogeny and identification of *Pantoea *species associated with plants, humans and the natural environment based on multilocus sequence analysis (MLSA) Syst Appl Microbiol 2008 31 6-8 447 460 10.1016/j.syapm.2008.09.004 19008066

[B31] Brady CL Venter SN Cleenwerck I Engelbeen K Vancanneyt M Swings J Coutinho TA *Pantoea vagans *sp. nov., *Pantoea eucalypti *sp. nov., *Pantoea deleyi *sp. nov. and *Pantoea anthophila *sp. nov Int J Syst Evol Microbiol 2009 59 Pt 9 2339 2345 10.1099/ijs.0.009241-0 19620357

[B32] Sambrook J Fritsch E Maniatis T Molecular cloning: a laboratory manual 1989 2 New York: Cold Spring Harbor Laboratory Press

[B33] Albert TJ Norton J Ott M Richmond T Nuwaysir K Nuwaysir EF Stengele KP Green RD Light-directed 5'-->3' synthesis of complex oligonucleotide microarrays Nucleic Acids Res 2003 31 7 e35 10.1093/nar/gng035 12655023 PMC152820

[B34] Nuwaysir EF Huang W Albert TJ Singh J Nuwaysir K Pitas A Richmond T Gorski T Berg JP Ballin J Gene expression analysis using oligonucleotide arrays produced by maskless photolithography Genome Res 2002 12 11 1749 1755 10.1101/gr.362402 12421762 PMC187555

[B35] Wang X He H Li L Chen R Deng XW Li S NMPP: a user-customized NimbleGen microarray data processing pipeline Bioinformatics 2006 22 23 2955 2957 10.1093/bioinformatics/btl525 17038341

[B36] Edgar R Domrachev M Lash AE Gene Expression Omnibus: NCBI gene expression and hybridization array data repository Nucleic Acids Res 2002 30 1 207 210 10.1093/nar/30.1.207 11752295 PMC99122

