# Supplementary material for: Broad spectrum microarray for fingerprint-based bacterial species identification
Source: BMC Biotechnol. 2010 Feb 17;10:13. doi: 10.1186/1472-6750-10-13 (PMC2830168; doi:10.1186/1472-6750-10-13)
Supplement: Additional file 1 — Pair-wise correlation coefficients for hybridisation patterns obtained with 10,895 probes corresponding to E. coli K12 perfect matches. [file 1472-6750-10-13-S1.DOC]

Supplementary Table 1: Pair-wise correlation coefficients for hybridisation patterns obtained with 10,895 probes corresponding to *E. coli* K12 perfect matches. Spearman’s rank correlation coefficients (top right) and difference between mean of replicate pair-wise correlations of one isolate to mean of non replicate pair-wise correlations of other isolate, both reciprocal values are indicated below each other (down left). Values from the same genera are highlighted in light grey and replicates from the same strains with a darker grey. Correlations can be higher between samples from different strain or species (indicated in italics) than between replicates. P. aggl represents *P. agglomerans*, P.vag for *P. vagans*, P. stew for *P. stewartii* subsp.  *indologenes*, and S. Typh for *Salmonella* Typhimurium, Xarbju for *X*. *arboricola* pv. *juglandis*, Xarbpr for *X. arboricola* pv. *pruni*, Xcamca for *X. campestris* pv. *campestris* and Xtratr for *X. translucens* pv. *translucens*. Replicates are indicated from a to d

Supplementary Table 1:

|  | **E.coliB-b** | **E.coliK12-a** | **E.coliK12-b** | **E.coliK12-c** | **E.coliK12-d** | **P.aggl27155-a** | **P.aggl27155-b** | **P.vagC9-1-a** | **P.vagC9-1-b** | **P.vagC9-1w-a** | **P.vagC9-1w-b** | **P.stew-a** | **P.stew-b** | **P.stew-c** | **S.TyphDT204-a** | **S.TyphDT204-b** | **S.TyphDT204-c** | **S.TyphLT2-a** | **S.TyphLT2-b** | **M.luteus-a** | **M.luteus-b** | **X.arbju-a** | **X.arbju-b** | **X.arbpr-a** | **X.arbpr-b** | **X.camca-a** | **X.camca-b** | **X.tratr-a** | **X.tratr-b** |
| --- | --- | --- | --- | --- | --- | --- | --- | --- | --- | --- | --- | --- | --- | --- | --- | --- | --- | --- | --- | --- | --- | --- | --- | --- | --- | --- | --- | --- | --- |
| **E.coliB-a** | **0.96** | **0.93** | **0.96** | **0.94** | **0.95** | **0.93** | **0.93** | **0.90** | **0.92** | **0.92** | **0.90** | **0.93** | **0.92** | **0.94** | **0.94** | **0.93** | **0.92** | **0.89** | **0.89** | **0.89** | **0.87** | **0.94** | **0.93** | **0.92** | **0.91** | **0.91** | **0.92** | **0.91** | **0.92** |
| **E.coliB-b** |  | **0.96** | ***0.97*** | ***0.97*** | ***0.97*** | **0.96** | ***0.97*** | **0.95** | **0.96** | **0.95** | **0.94** | **0.96** | **0.96** | **0.96** | **0.96** | ***0.97*** | **0.96** | **0.94** | **0.94** | **0.89** | **0.87** | **0.94** | **0.94** | **0.93** | **0.93** | **0.94** | **0.95** | **0.94** | **0.95** |
| **E.coliK12-a** |  |  | **0.97** | **0.98** | **0.98** | **0.96** | **0.97** | **0.97** | **0.97** | **0.95** | **0.95** | **0.96** | **0.96** | **0.97** | **0.96** | **0.96** | **0.96** | **0.96** | **0.96** | **0.90** | **0.87** | **0.92** | **0.94** | **0.92** | **0.92** | **0.95** | **0.96** | **0.94** | **0.95** |
| **E.coliK12-b** | **0.01** |  |  | **0.98** | **0.98** | **0.96** | **0.96** | **0.94** | **0.95** | **0.94** | **0.93** | **0.95** | **0.95** | **0.96** | **0.96** | **0.97** | **0.96** | **0.94** | **0.93** | **0.90** | **0.88** | **0.94** | **0.94** | **0.93** | **0.92** | **0.95** | **0.96** | **0.94** | **0.95** |
| **E.coliK12-c** | **0.02** |  |  | **0.98** | **0.99** | **0.97** | **0.98** | **0.97** | **0.97** | **0.96** | **0.95** | **0.97** | **0.97** | **0.97** | ***0.97*** | **0.97** | **0.96** | **0.96** | **0.95** | **0.90** | **0.88** | **0.94** | **0.95** | **0.93** | **0.93** | **0.96** | **0.97** | **0.95** | **0.96** |
| **E.coliK12-d** |  |  |  |  |  | **0.97** | **0.97** | **0.96** | **0.97** | **0.95** | **0.94** | **0.96** | **0.97** | **0.97** | **0.96** | **0.96** | **0.96** | **0.95** | **0.95** | **0.89** | **0.87** | **0.94** | **0.94** | **0.93** | **0.92** | **0.95** | **0.96** | **0.94** | **0.95** |
| **P.aggl27155-a** | **0.02** |  |  | **0.01** |  |  | **0.98** | **0.97** | **0.97** | **0.97** | **0.95** | **0.97** | ***0.98*** | **0.98** | **0.95** | **0.95** | **0.96** | **0.96** | **0.96** | **0.88** | **0.87** | **0.92** | **0.93** | **0.92** | **0.91** | **0.94** | **0.96** | **0.94** | **0.95** |
| **P.aggl27155-b** | **0.03** |  |  | **0.02** |  |  |  | **0.98** | **0.98** | **0.97** | **0.96** | **0.97** | ***0.98*** | ***0.99*** | **0.96** | **0.96** | **0.96** | **0.97** | **0.97** | **0.90** | **0.88** | **0.93** | **0.94** | **0.93** | **0.92** | **0.96** | **0.97** | **0.95** | **0.96** |
| **P.vagC9-1-a** | **0.03** |  |  | **0.02** |  |  | **0.01** |  | **0.99** | **0.97** | ***0.98*** | **0.97** | **0.97** | **0.98** | **0.96** | **0.96** | **0.95** | **0.97** | **0.97** | **0.90** | **0.89** | **0.90** | **0.93** | **0.91** | **0.92** | **0.96** | **0.96** | **0.95** | **0.95** |
| **P.vagC9-1-b** | **0.06** |  |  | **0.03** |  |  | **0.01** |  |  | **0.97** | **0.97** | ***0.98*** | **0.97** | **0.98** | ***0.97*** | **0.97** | **0.96** | **0.97** | **0.96** | **0.89** | **0.87** | **0.92** | **0.94** | **0.92** | **0.93** | **0.95** | **0.96** | **0.94** | **0.95** |
| **P.vagC9-1w-a** | **0.04** |  |  | **0.03** |  |  | **0.02** |  | **0.02** |  | **0.97** | **0.97** | **0.96** | **0.97** | **0.96** | **0.95** | **0.96** | **0.95** | **0.94** | **0.91** | **0.90** | **0.93** | **0.94** | **0.94** | **0.94** | **0.96** | **0.96** | **0.95** | **0.95** |
| **P.vagC9-1w-b** | **0.04** |  |  | **0.02** |  |  | **0.01** |  | ***0.00*** |  |  | **0.96** | **0.95** | **0.97** | **0.96** | **0.95** | **0.94** | **0.96** | **0.95** | **0.91** | **0.90** | **0.91** | **0.94** | **0.92** | **0.93** | **0.96** | **0.96** | **0.95** | **0.95** |
| **P.stew-a** | **0.02** |  |  | **0.01** |  |  | **0.01** |  | **0.01** |  | **0.01** |  | **0.97** | **0.98** | ***0.97*** | **0.96** | **0.96** | **0.95** | **0.95** | **0.92** | **0.90** | **0.93** | **0.94** | **0.93** | **0.93** | **0.96** | **0.96** | **0.95** | **0.95** |
| **P.stew-b** | **0.03** |  |  | **0.01** |  |  | ***0.00*** |  | ***0.00*** |  | **0.01** |  | **0.98** | **0.98** | **0.95** | **0.96** | **0.96** | **0.97** | **0.97** | **0.88** | **0.86** | **0.92** | **0.93** | **0.92** | **0.91** | **0.95** | **0.96** | **0.94** | **0.95** |
| **P.stew-c** |  |  |  |  |  |  |  |  |  |  |  |  |  |  | ***0.97*** | **0.96** | **0.96** | **0.97** | **0.97** | **0.91** | **0.89** | **0.93** | **0.94** | **0.93** | **0.93** | **0.97** | **0.97** | **0.96** | **0.96** |
| **S.TyphDT204-a** | **0.02** |  |  | **0.02** |  |  | **0.03** |  | **0.03** |  | **0.01** |  | **0.02** |  |  | **0.97** | **0.96** | **0.95** | **0.94** | **0.92** | **0.90** | **0.94** | **0.95** | **0.94** | **0.94** | **0.96** | **0.96** | **0.95** | **0.95** |
| **S.TyphDT204-b** | **0.02** |  |  | **0.01** |  |  | **0.01** |  | **0.01** |  | **0.01** |  | **0.01** |  |  | **0.97** | **0.97** | **0.95** | **0.94** | **0.89** | **0.88** | **0.94** | **0.94** | **0.93** | **0.94** | **0.95** | **0.95** | **0.95** | **0.95** |
| **S.TyphDT204-c** |  |  |  |  |  |  |  |  |  |  |  |  |  |  |  |  |  | **0.95** | **0.94** | **0.89** | **0.89** | **0.94** | **0.94** | **0.93** | **0.94** | **0.95** | **0.96** | **0.95** | **0.96** |
| **S.TyphLT2-a** | **0.05** |  |  | **0.03** |  |  | **0.02** |  | **0.02** |  | **0.02** |  | **0.01** |  |  | **0.02** |  |  | **0.99** | **0.87** | **0.86** | **0.89** | **0.92** | **0.90** | **0.91** | **0.95** | **0.95** | **0.94** | **0.94** |
| **S.TyphLT2-b** | **0.07** |  |  | **0.04** |  |  | **0.03** |  | **0.02** |  | **0.04** |  | **0.03** |  |  | **0.04** |  |  |  | **0.86** | **0.84** | **0.88** | **0.91** | **0.89** | **0.89** | **0.94** | **0.95** | **0.93** | **0.93** |
| **M.luteus-a** | **0.09** |  |  | **0.09** |  |  | **0.10** |  | **0.10** |  | **0.06** |  | **0.08** |  |  | **0.07** |  |  | **0.13** |  | **0.96** | **0.90** | **0.93** | **0.92** | **0.91** | **0.95** | **0.94** | **0.94** | **0.94** |
| **M.luteus-b** | **0.08** |  |  | **0.07** |  |  | **0.08** |  | **0.07** |  | **0.06** |  | **0.07** |  |  | **0.06** |  |  | **0.10** |  |  | **0.90** | **0.92** | **0.91** | **0.91** | **0.94** | **0.93** | **0.94** | **0.93** |
| **X.arbju-a** | **0.03** |  |  | **0.04** |  |  | **0.06** |  | **0.07** |  | **0.04** |  | **0.05** |  |  | **0.03** |  |  | **0.09** |  | **0.05** |  | **0.97** | **0.95** | **0.95** | **0.94** | **0.94** | **0.93** | **0.95** |
| **X.arbju-b** | **0.05** |  |  | **0.03** |  |  | **0.04** |  | **0.05** |  | **0.04** |  | **0.04** |  |  | **0.02** |  |  | **0.07** |  | **0.05** |  |  | ***0.96*** | ***0.97*** | **0.97** | **0.96** | **0.97** | **0.97** |
| **X.arbpr-a** | **0.04** |  |  | **0.05** |  |  | **0.06** |  | **0.07** |  | **0.04** |  | **0.05** |  |  | **0.03** |  |  | **0.09** |  | **0.05** |  | **0.01** |  | **0.95** | **0.95** | **0.95** | **0.94** | **0.95** |
| **X.arbpr-b** | **0.03** |  |  | **0.03** |  |  | **0.03** |  | **0.03** |  | **0.02** |  | **0.02** |  |  | **0.01** |  |  | **0.05** |  | **0.04** |  | ***-0.01*** |  |  | **0.96** | **0.95** | **0.95** | **0.96** |
| **X.camca-a** | **0.03** |  |  | **0.02** |  |  | **0.03** |  | **0.03** |  | **0.01** |  | **0.02** |  |  | **0.01** |  |  | **0.04** |  | **0.02** |  | **0.02** |  | ***0.00*** |  | **0.99** | **0.98** | **0.98** |
| **X.camca-b** | **0.06** |  |  | **0.03** |  |  | **0.03** |  | **0.03** |  | **0.03** |  | **0.03** |  |  | **0.03** |  |  | **0.04** |  | **0.05** |  | **0.04** |  | **0.04** |  |  | **0.98** | **0.98** |
| **X.tratr-a** | **0.04** |  |  | **0.03** |  |  | **0.04** |  | **0.04** |  | **0.02** |  | **0.03** |  |  | **0.02** |  |  | **0.06** |  | **0.02** |  | **0.01** |  | ***0.00*** |  | **0.01** |  | **0.98** |
| **X.tratr-b** | **0.06** |  |  | **0.04** |  |  | **0.04** |  | **0.04** |  | **0.04** |  | **0.03** |  |  | **0.03** |  |  | **0.05** |  | **0.05** |  | **0.03** |  | **0.03** |  | ***0.00*** |  |  |
